# A Pipeline for 3D Multimodality Image Integration and Computer-assisted Planning in Epilepsy Surgery

**DOI:** 10.3791/53450

**Published:** 2016-05-20

**Authors:** Mark Nowell, Roman Rodionov, Gergely Zombori, Rachel Sparks, Michele Rizzi, Sebastien Ourselin, Anna Miserocchi, Andrew McEvoy, John Duncan

**Affiliations:** ^1^Department of Clinical and Experimental Epilepsy, UCL Institute of Neurology; ^2^Center of Medical Imaging and Computing, UCL; ^3^Department of Neurosurgery, National Hospital for Neurology and Neurosurgery

**Keywords:** Medicine, Issue 111, epilepsy, surgery, multimodality, imaging, 3D, planning

## Abstract

Epilepsy surgery is challenging and the use of 3D multimodality image integration (3DMMI) to aid presurgical planning is well-established. Multimodality image integration can be technically demanding, and is underutilised in clinical practice. We have developed a single software platform for image integration, 3D visualization and surgical planning. Here, our pipeline is described in step-by-step fashion, starting with image acquisition, proceeding through image co-registration, manual segmentation, brain and vessel extraction, 3D visualization and manual planning of stereoEEG (SEEG) implantations. With dissemination of the software this pipeline can be reproduced in other centres, allowing other groups to benefit from 3DMMI. We also describe the use of an automated, multi-trajectory planner to generate stereoEEG implantation plans. Preliminary studies suggest this is a rapid, safe and efficacious adjunct for planning SEEG implantations. Finally, a simple solution for the export of plans and models to commercial neuronavigation systems for implementation of plans in the operating theater is described. This software is a valuable tool that can support clinical decision making throughout the epilepsy surgery pathway.

**Figure:**
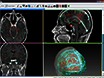


## Introduction

In surgical practice it is crucial for the surgeon to appreciate anatomical structures and their spatial relationships to one another in three dimensions. This is especially important in neurosurgery, where the surgeon is working in a confined space, with limited visualization and access to complex anatomy. Despite this, to date most imaging has been presented to surgeons in conventional planar 2D form, and different imaging modalities are often presented one after another in series. As a consequence, the surgeon has to mentally integrate this data for each patient, and place it into an anatomical framework for presurgical planning. There is clear benefit in generating 3D computer models of the individual patient brain, which demonstrates the anatomy of the cortex, the blood vessels, any pathological lesions present as well as other relevant 3D landmarks in the same spatial context^1-4^. Before surgery the surgeon can rotate and alter the transparency of these models, to fully understand the 3D relationships between different structures of interest. This principle is termed 3D multimodality imaging (3DMMI).

The aim of pre-surgical evaluation for epilepsy surgery is to infer the localization of the area of the brain where seizures arise, and ensure that this can be safely resected without causing significant deficits^5^. There is a wide range of diagnostic imaging modalities that contribute to this, including structural MRI, fluorodeoxyglucose positron emission tomography (FDG-PET), ictal single photon emission computed tomography (SPECT), magnetoencephalography (MEG) dipoles, functional MRI (fMRI) and diffusion tensor imaging (DTI)^6^. Epilepsy surgery is ideally suited for 3DMMI since it requires the simultaneous interpretation of multiple data sets, and the consideration of how each data set relates to another.

In many cases non-invasive investigations fail to provide the level of evidence required to proceed to resection. In these cases intracranial EEG (IC EEG) recordings are necessary to identify the region of the brain that must be removed to prevent seizures. Increasingly, IC EEG is performed by a technique called SEEG, in which a number of recording depth electrodes are placed intracerebrally to capture the origin and propagation of electrical activity associated with seizures in 3D^1,7-10^.

The first step of SEEG implantations is to develop the strategy of the implantation, defining the areas of the brain that need to be sampled. This involves integrating the clinical and non-invasive EEG date, with structural imaging, with any lesion, and functional imaging data that infer the location of the source of the epilepsy.

The second step is the precise surgical planning of the electrode trajectories. The surgeon must ensure safe avascular electrode trajectories, centring electrode entries at the crown of the gyri and remote from cortical surface veins, and traversing the skull orthogonally. Additionally the entire implantation arrangement has to be well conceived, with reasonable inter-electrode spacing and no electrode collisions.

The feasibility of generating 3DMMI models to guide implantation of IC EEG electrodes in a busy epilepsy surgery practice has previously been demonstrated^11^. We have also demonstrated the principle that the use of 3DMMI confers added value in clinical decision-making. In a prospective study, disclosure of 3DMMI changed some aspect of management in 43/54 (80%) cases, and specifically changed the positioning of 158/212 (75%) of depth electrodes^12^.

There is a range of software packages that facilitate 3DMMI. These include commercially available neuronavigation platforms that are used in the operating theater, specialised planning software suites allied with neuronavigation platforms and research-orientated stand-alone image integration and visualization platforms. As the functionality, flexibility and versatility of these platforms increase, the usability and likelihood of translating them into clinical practice correspondingly decreases.

We have developed custom-designed software for multimodality image integration, advanced 3D visualization and SEEG electrode placement planning^12,13^ for the treatment of epilepsy. The emphasis is on ease of use in a clinical scenario, allowing real time use of software by clinicians, and rapid incorporation into the clinical pipeline. The software runs on a translational imaging platform^14^, that combines NiftyReg, NiftySeg and NiftyView.

In this paper the protocol for using the software in clinical practice is set out. The steps for image co-registration, segmentation of regions of interest, brain segmentation, extracting blood vessels from dedicated vascular imaging^15^, building 3D models, planning SEEG implantations and rapidly exporting models and plans to the operating theater are described. A novel tool is also described for automated multi-trajectory planning^13^, that increases the safety and efficacy of the implantations and substantially reduces the duration of planning.

## Protocol

**NOTE: Software commands provided here are specific to the current version (19.01.2015) of the software and may change upon subsequent software releases. Manuals for individual versions are available on request.**

### 1. Perform Image Integration and Visualization

1. Acquire imaging.
  1. Acquire neuronavigation T1-weighted MRI scan with gadolinium enhancement- this will be the reference image. (Note: Image acquisition requirements are available from the neuronavigation commercial supplier^11,12^. See **Table 1**).
  2. Collect all other imaging done during presurgical evaluation in DICOM or Nifti format (may include functional MRI (fMRI), diffusion tensor imaging (DTI) tractography, fluoro-deoxyglucose positron emission tomography (FDG-PET), ictal-interictal single photon emission CT (SPECT), magnetoencephalography (MEG) dipole, 3D phase contrast MRI, CT angiography) See **Table 1**.
2. Run pre-processing outside in-house software.
  1. Process isometric T1 weighted MRI with open source Freesurfer software, run on a linux workstation using the command 'recon-all', to generate cortical segmentations.
  2. Convert wmparc.mgz and ribbon.mgz files to nifti format using the command 'mrconvert'
3. Open in-house software on Windows PC and load data (**Figure 1**).
  1. Note 2 x 2 window display, DataManager on far left, icons on top representing different image processing tools and selected tool on far right.
  2. Import data using 'drag and drop', by accessing the main menu "File/Open" or by a speed button (icon) "Open". Scroll through different data sets to ensure completeness. Note the zoom function by right clicking and moving mouse, and the hierarchical nature of DataManager, with consecutive image overlay.
4. Coregister images.
  1. Single images.
    1. Select NiftyReg tool from speed icons.
    2. Select neuronavigation T1 with gadolinium in DataManager- this will be reference image that all other imaging is coregistered to.
    3. Select 'floating image' to be coregistered to reference image.
    4. Define name and location of registered image. Set optimization parameters to level number 4, level to perform 3, iteration number 5, coregistration type rigid body.
    5. Run automated rigid body coregistration by clicking on 'Run' button.
    6. Check accuracy of coregistration. Inspect the registered image over the reference image, and alter transparency of registered image by right-clicking on the image in DataManager, and moving the 'Opacity' cursor. Verify coregistration by inspecting clear anatomical landmarks such as the foramen of Monroe.
  2. Paired images.
    1. Coregister 'space-defining image' first (eg. Fractional anisotropic map), using the NiftyReg tool as in steps 1.4.1.1 - 1.4.1.6.
    2. Select RegResample tool from speed icons.
    3. Select neuronavigation T1 with gadolinium in DataManager as reference image.
    4. Select image with results of processing (*e.g*., tractography image) as floating image.
    5. Use txt file generated from previous registration of 'space-defining image' as input transformation.
    6. Define name and location of registered image. Select interpolation type as 0.
    7. Run resampling of 'result of processing' by clicking on 'Run' button.
    8. View new generated image by selecting in DataManager
    9. Check accuracy of coregistration as in step 1.4.1.6.
  3. Repeat steps 1.4.1 - 1.4.2 for all data sets.
5. Segment images.
  1. Select image to be segmented in DataManager, and select Segmentation Editor tool from speed icons.
  2. Use advanced segmentation tools (manual segmentation, region-growing, subtracting) to draw region of interest on several slices of imaging in axial, coronal and sagittal planes
  3. Select 3D interpolation to visualise evolving segmented structure in 3D window. Confirm segmentation to generate new Nifti file of segmented structure.
  4. Repeat steps 1.5.1 - 1.5.3 for all images where manual segmentation is indicated.
6. Generate brain models.
  1. Select wmparc.nii image on DataManager, and ensure wmparc.nii is coregistered with reference image using steps 1.4.1.
  2. Select Basic Processing Tools from speed icons.
  3. Apply a threshold to wmparc.nii from 1-5002 to create binarised mask of cortex.
7. Render regions of interest as 3D surfaces (**Figure 2, 3**). Note: Visualization of data sets as 3D surface renderings (stl files) can be done in two ways:
  1. Use Surface Extractor tool.
    1. Select Surface Extractor icon. Define threshold for surface extraction and select Apply. Name surface rendering in DataManager.
  2. Right click on Nifti file in DataManager and select' Smooth Polygon Surface'.
8. Extract surface models of vessels (**Figure 4**). Note: Extracting vessels from dedicated vascular imaging (3D phase contrast MRI, CT angiography, T1 weighted MRI with gadolinium) can be done in two ways.
  1. Use Surface Extractor tool.
    1. Coregister the vascular imaging to the reference image using NiftiReg. 3D surface render the image using Surface Extractor.
    2. Generate intracranial mask by applying the dilation and closing functions in Basic Image Processing to the binarised mask of cortex. Apply intracranial mask to the vascular imaging using the multiply function in Basic Image Processing to remove extracranial vessels.
    3. Remove noise from the stl file by processing outside in-house software, using 3D mesh processing software package. Note: Instructions for use of this tool are freely available online.
  2. Use VesselExtractor tool.
    1. Select the VesselExtractor tool from the speed icons. Select vascular image data set and specify the name and location of the vessel-extraction Nifti file.
    2. Run VesselExtractor by clicking on 'Run'. Apply intracranial mask to the results of VesselExtractor using the multiply function in Basic Image Processing to remove extracranial vessels. Note: Intracranial mask generated by applying the dilation and closing functions in Basic Image Processing to the binarised mask of cortex as in 1.8.1.2.
  3. Repeat process of 1.8.1 or 1.8.2 for CT angiography, 3D phase contrast MRI and neuronavigation T1 with gadolinium.
9. Generate volume rendering of brain (**Figure 5**).
  1. Select wmparc.nii image on DataManager, and ensure wmparc.nii is coregistered with reference image using steps 1.4.1.
  2. Select Basic Processing Tools from speed icons.
  3. Apply Gaussian smoothing to wmparc.nii image, using Basic Processing tools.
  4. Select volume rendering tool from speed icons, and ensure smoothed wmparc.nii file is highlighted in DataManager.
  5. Tick 'volume rendering' box inside volume rendering tool to generate volume rendering of cortex.

### 2. Perform Manual Planning

1. Use Trajectory Planner speed icon.
  1. Select neuronavigation T1 scan as the reference image. Select New Plan, and New trajectory.
  2. Select target point on planar imaging by pressing 'Alt' and right-click on the mouse, based on list of desired anatomical target points by clinicians. Note: examples of targets include mesial temporal structures (amygdala, hippocampus), insula, cingulate gyrus.
  3. Select entry point on planar imaging by pressing 'Alt' and left-click on the mouse, based on the list of desired entry points by clinicians. Note: examples of entry points include middle temporal gyrus, precentral gyrus, supramarginal gyrus.
  4. Observe linear trajectory generated between target and entry point.
2. Visualise risk.
  1. Select Risk Visualization speed icon to examine trajectory length.
  2. Select 'Link view planes' to link Probes eye viewer to orthogonal view planes in main window.
  3. Scroll along trajectory, examining Probes eye viewer to ensure avascular path.

### 3. Perform Computer-assisted Planning

1. Prepare data.
  1. Prepare grey matter surface.
    1. Select the ribbon.nii file generated from cortical segmentation software.
    2. Co-register ribbon.nii file to reference image using NiftiReg.
    3. 3D surface render co-registered image by using 'Smooth polygon surface' function.
  2. Prepare scalp and scalp exclusion template.
    1. Select T1 neuronavigation image as reference image.
    2. Use Basic Image Processing tool to apply Gaussian transformation.
    3. Surface render image using Surface Extractor, to generate scalp surface.
    4. Save and export image as stl file.
    5. Load stl fle into 3D mesh processing software.
    6. For scalp, use cleaning and editing tools to delete intracranial contents.
    7. For scalp exclusion template, use manual editing tools to remove areas not appropriate for electrode entry points (*i.e*., face, ears, contralateral hemisphere, area below tentorium cerebelli).
  3. Prepare surface sulci surface.
    1. Generate whole sulci.
      1. Binarise wmparc.nii file using the Basic Image Processing tool as in step 1.6.3.
      2. Close binarised wmparc.nii file by 3 using Basic Image Processing tool.
      3. Subtract binarised file generated in 3.1.3.1.1 from closed binarised file generated in 3.1.3.1.2 using Basic Image Processing tool.
    2. Remove the sulci at depth to generate the surface sulci image. Note: using surface sulci image as a critical structure has the advantage of spacing trajectories away from sulci at the surface of the brain, and permitting trajectories to approach sulci at depth, which is where grey matter lies.
      1. Reduce the closed, binarised wmparc file generated in 3.1.3.1.2 using the Basic Image Processing tool.
      2. Invert file generated in 3.1.3.2.1 using the Basic Image Processing tool.
      3. Multiply file generated in 3.1.3.2.2 by the whole sulci generated in 3.1.3.1.3, using the Basic Image Processing tool.
2. Run multi-trajectory planner (**Figure 6**). NOTE: Automated multi-trajectory planning is dependent on robust data preparation; surface renderings of the scalp, scalp exclusion mask, intracranial vasculature, surface sulci, cortex and grey matter are required.
  1. Select Trajectory Planner from speed icons. Select reference image as neuronavigation T1 MRI.
  2. Select 'target points'; multiple target points can be entered by 'Shift' and mouse left-click, or by loading a saved target point set. Note: examples of target include mesial temporal structures (amygdala, hippocampus), insula, cingulate gyrus.
  3. Select 'entry points', and select scalp exclusion mask on the attached dropdown menu. Note: This has the purpose of restricting the search of possible entry points to a restricted area that is surgically feasible to implement.
  4. Select critical structures, marking the surfaces from the dropdown list that the trajectories should avoid. Select advanced settings; adjust the user-defined constraints regarding trajectory length, angle of entry and distance between trajectories as preferred.
  5. Select grey matter- white matter evaluation and stratify risk sort to optimise the proportion of the trajectories that lie in grey matter.
  6. Run multi-trajectory planner by selecting Add New Plan, and Recompute Plan.
3. Visualise risk (**Figure 7**).
  1. Assess risk and safety profiles after trajectory planning, using the Risk Visualization speed icon. Note: For each trajectory there are metrics for length, angle of entry, cumulative risk, minimum distance to blood vessel and grey matter white matter ratios, plus graphic representation along the trajectory path of distance to critical structures. A probes eye viewer is also included.
  2. Select Risk map in DataManager to show a colour-coded contour map overlying the scalp exclusion mask, with potential entry points represented and the associated level of risk colour coded, with red representing high risk and green representing low risk for any selected trajectory.
4. Manual adjustment of trajectories.
  1. Select trajectory.
  2. Select new entry point by pressing Alt and mouse right-click, and new target point by pressing Alt and mouse left-click.
  3. Assess new trajectory using the Risk Visualization speed icon as in step 3.3.

### 4. Export Plans and Models to the Operating Theater

1. Check that reference image is in DICOM format. Select S7 Export from speed icon.
2. Define the reference image, the plans and trajectories and the models that are to be exported, and specify the destination of the saved archive. Run S7 export tool.
3. Upload generated archive onto a USB stick for transfer to a neuronavigation system in the operating theater, and load archived folder on the neuronavigation system for clinical implementation of planned trajectories.

### 5. Reconstructing Electrode Implantation Post-operatively

1. Acquire post-operative CT imaging.
2. Load CT head onto in-house software, and load previously saved patient data set.
3. Coregister CT to reference T1 weighted MRI using the NiftyReg tool.
4. Generate 3D surface rendering of electrodes using the SurfaceExtractor tool on the registered CT, with high thresholding.
5. Clean surface rendered electrodes of noise, and wires using the cleaning and repair functions of 3D mesh processing software.

## Representative Results

The protocol described for image integration, visualization, manual planning and export to a selected neuronavigation system has been employed at the National Hospital for Neurology and Neurosurgery since August 2013. This comprises 35 cases of SEEG implantation^12^, with the implantation of 319 depth electrodes. 27/35 (77%) of patients have progressed to a cortical resection following implantation, which is an indicator that the implantation identified the area of seizure onset. There has been one haemorrhagic complication related to the placement of depth electrodes, and this was treated conservatively.

The imaging modalities used during the presurgical evaluation are decided on a case-by case basis, and are described in **Table 1**. The protocol is flexible, and can incorporate any imaging modality that can be imported into DICOM or Nifti format. **Figure 1** demonstrates the basic viewer for our in-house software platform, and **Figures 2, 3, 4 and 5** illustrate typical screenshots during the building of the 3D multimodality models.

The seamless integration of this protocol into our clinical pipeline, and the dissemination of this software to other centres, is a useful surrogate 'marker' of success. The difficulties in assessing clinical benefit in the epilepsy surgery population are well known and described elsewhere^12^. This pipeline offers a streamlined solution, which is flexible, relatively user-friendly, and easy to replicate in other centres.

Computer-assisted planning (CAP) is a recent development that has been retrospectively tested on previous manually planned implantations^16^. Preliminary results suggest that CAP generates safer, more efficient implantations, that are feasible to implement and that are completed in a time effective manner^16.^ **Table 2** demonstrates this quantitative comparison. A prospective trial of using CAP in clinical practice is underway. The algorithm that drives CAP has been previously described^13^.

**Figure 6** shows a typical outcome from the automated multi-trajectory planner. The critical structures that have been entered are veins, arteries and surface sulci. Note the centring of the trajectories on the crown of the gyri, and the constraining of trajectory entry points to a scalp exclusion mask. **Figure 7** shows a typical risk visualization graph for an individual trajectory, with associated metrics and graphic representation of trajectory length.


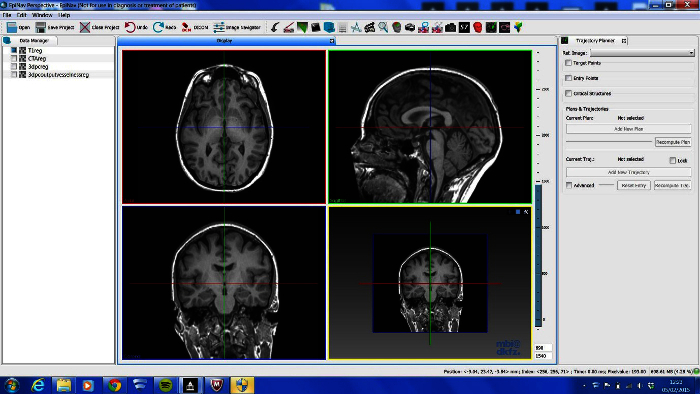
**Figure 1. Basic Viewer Display of In-house Software Platform**. LEFT- DataManager, TOP- toolbar that contains shortcuts plug-in tools, RIGHT- current plug in tool in use, CENTRE- 4 Ortho-view display. Please click here to view a larger version of this figure.


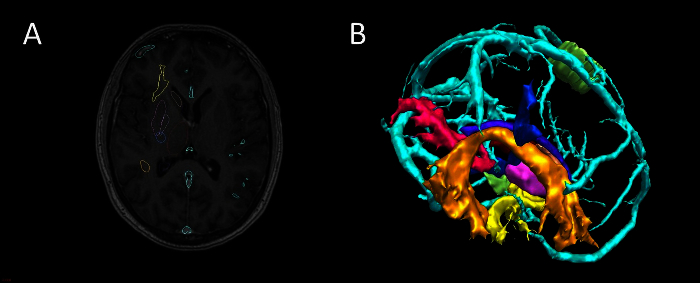
**Figure 2. Segmentation and 3D Visualization in In-house Software**. (**A**) axial T1 MRI with superimposed surface models, (**B**) 3D surface rendering of models (cyan-veins, green- motor hand from transcranial magnetic stimulation, orange- arcuate fasciculus tractography, blue- corticospinal tractography, pink- optic radiation tractography, yellow- uncinate fasciculus tractography, purple- thalamus segmentation). Please click here to view a larger version of this figure.


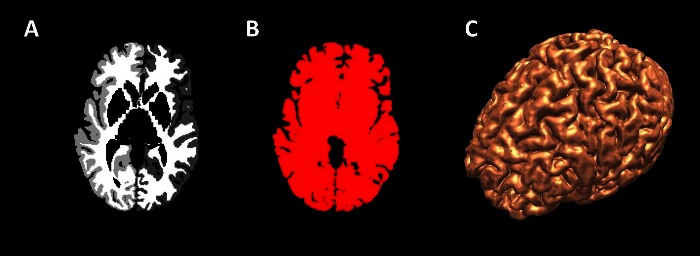
**Figure 3. Generation of Cortex Surface Models**. (**A**) axial view of wmparc file, (**B**) wmparc file thresholded from 1-5002, (**C**) surface rendering of binarised wmparc file. Please click here to view a larger version of this figure.


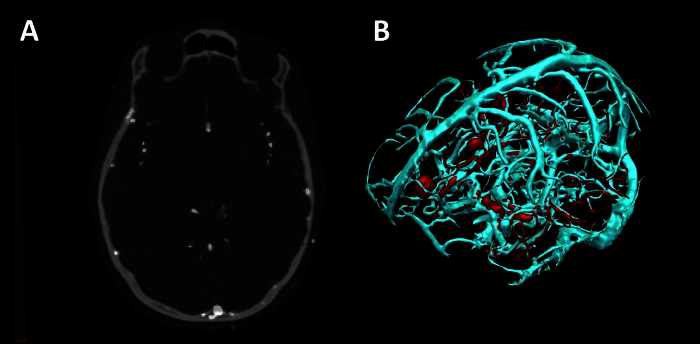
**Figure 4**. **Vessel Extraction in In-house Software using Vesselness**. (**A**) Axial CT angiogram co-registered with 3D phase contrast MRI. (**B**) 3D surface rendering of veins (cyan) and arteries (red). Please click here to view a larger version of this figure.


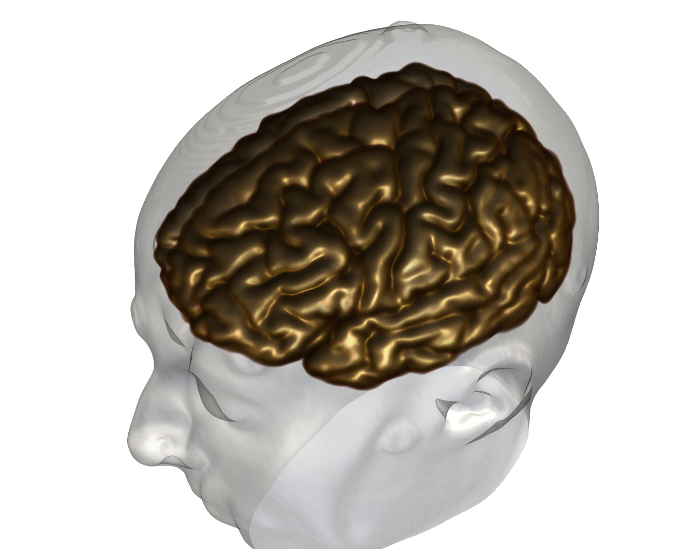
**Figure 5. Generation of Cortex Volume Model **3D Volume rendering of cortex (grey) and surface rendering of scalp surface (white). Please click here to view a larger version of this figure.


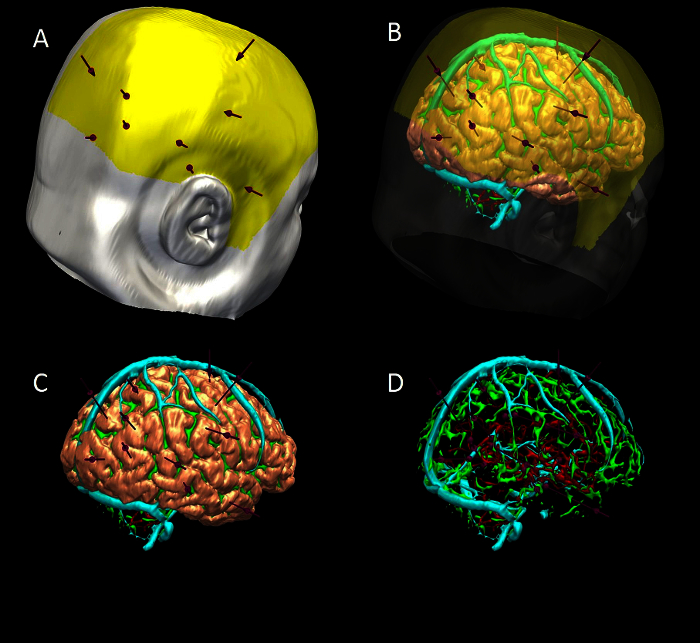
**Figure 6. 3D Multimodality Models of Computer-assisted Trajectory Planning**. (**A**) scalp (white), scalp exclusion mask (yellow) and trajectories (purple). (**B**) scalp and mask transparent to show brain (pink), sulci (green), veins (cyan) and arteries (red). (**C**) scalp and mask removed to show trajectories and brain. (**D**) brain removed to show trajectories , surface sulci, veins and arteries. Please click here to view a larger version of this figure.


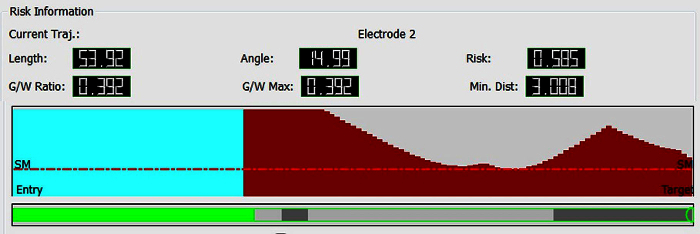
**Figure 7**. **Graphic Visualization of Metrics Associated with Individual Trajectories**. Top- length, angle traversing skull, risk, G/W ratio and minimum distance from a blood vessel >1 mm in diameter. Middle- graphic display of closest critical structure along length of trajectory (red-artery, cyan-vein, y-axis- distance to structure(maximum 10 mm), x-axis- distance along trajectory from brain entry to target, SM- safety margin represented as horizontal red line that marks 3 mm separation of trajectory to critical structure). Bottom- graphic display of trajectory path through grey and white matter (green-extracerebral, grey-grey matter, white- white matter). Please click here to view a larger version of this figure.

**Table:** 

**Modality**	**Site**	**Pre-processing**	**Field of view (AP x RL x IS)**	**Voxel size (AP x RL x IS)**
3D T1 FSPGR	ES	No	256 x 256 x 166	0.94 x 0.94 x 1.1
Coronal T2 FLAIR	ES	No	256 x 160 x 32	0.94 x 1.5 x 3.5
Navigation T1 with gadolinium	NHNN	No	512 x 512 x 144	0.5 x 0.5 x 1.5
MRI 3D phase contrast	NHNN	No	256 x 256 x 160	0.85 x 0.85 x 1
CT angiogram	NHNN	No	512 x 512 x 383	0.43 x 0.43 x 0.75
MEG dipole	NHNN	Yes		
Ictal-interictal SPECT	UCLH	Yes	128 x 128 x 49	3.9 x 3.9 x 3.9
FDG-PET	UCLH	Yes	128 x 128 x 47	1.95 x 1.95 x 3.3
DTI	ES	Yes	128 x 128 x 60	1.88 x 1.88 x 2.4
Functional MRI, EEG-correlated fMRI	ES	Yes	128 x 128 x 58	1.87 x 1.87 x 2.5

**Table 1.Imaging Modalities used for Image Integration** ((ES-Epilepsy Society, NHNN-National Hospital for Neurology and Neurosurgery, UCLH- University College London Hospital, FSPGR-FastSpoiledGradientRecalledEcho, MEG-magnetoencephalography, SPECT-single photon emission computed tomography, FDG PET- fluorodeoxyglucose positron emission tomography, DTI-diffusion tensor imaging, AP- anterior posterior, RL - right left, IS - inferior superior).

**Table:** 

	Manual Planning*	CAP*	**Estimated Difference (Manual-CAP)**	**Error**	**P value**
**Electrode Length (mm, 1 dp)**	57.9 (21.8)	53.9 (15.6)	4.74	1.59	<0.05
**Angle of Entry (degrees off perpendicular, 1 dp)**	16.2 (12.8)	13.0 (7.6)	5.89	1.07	<0.05
**Risk (normalised units, 2 dp)**	0.41 (0.79)	0.36 (0.42)	0.19	0.03	<0.05
**Minimum Distance from Blood Vessel (mm, 1 dp)**	4.5 (3.0)	4.5 (3.0)	-0.56	0.2	<0.05
**Proportion of Intracerebral Electrode in Grey Matter (2 dp)**	0.33 (0.33)	0.48 (0.28)	-0.11	0.02	<0.05

**Table 2.Statistical Comparison between Manual and Computer-assisted Planning (CAP).** *first value is median, second value in brackets is interquartile range. This Table has been reproduced with permission from ^16^.

## Discussion

In summary, the crucial steps for image integration and 3D visualization are image co-registration, segmentation of brain, vessels and other structures or areas of interest, and export to a neuronavigation system. This process was previously performed in the group using commercially available image integration software. A disadvantage to this pipeline was the time taken, with the entire process taking 2 - 4 hr. Using our in-house software platform, this pipeline is simplified considerably, and can be completed in 1 - 2 hr. Further, there is the added functionality of surgical planning of SEEG electrode trajectories on this software, that can be done manually or with computer-assistance. The benefits of CAP over manual planning are increased precision, reduced risk and increased speed, and have been discussed elsewhere (Nowell *et al*, In Press, Sparks *et al*, submitted).

The in-house software platform is in continuous development, with new tools and functionality being added to support all stages of presurgical evaluation and surgical management. There is therefore a need for rigorous testing at each new version release. Current limitations of the software include a lack of high quality volume rendering, which is present in other platforms and is a valuable addition for advanced 3D visualization. Also export is only compatible with a selected neuronavigation company at the present time. These limitations have not affected the clinical utility of the software in our unit, and have not slowed the dissemination of the technology to other centres.

The significance of this software is that it removes the barriers that previous groups have cited as reasons for not using 3DMMI. The solution provides easy to use tools in one single platform, that does not require specialist training or expertise, is time and cost-effective and is easily translated into clinical practice. We have plans to add further innovations to the software to support Epilepsy Surgery. Furthermore, the methods could easily be applied to other areas of neurosurgery, such as resection of low grade tumours close to eloquent cortex, focal lesioning and delivery of targeted stimulation. 3DMMI and precise surgical planning tools are likely to become increasingly important in modern surgery, as more challenging cases are taken on and as minimally invasive treatments enter common practice.

## Disclosures

Funding: Mark Nowell, Gergely Zombori, Rachel Sparks and Roman Rodionov are supported by the Department of Health and Wellcome Trust through the Health Innovation Challenge Fund (HICF-T4-275, Programme Grant 97914).

John Duncan has received Institutional grant support from Eisai, UCB Pharma, GSK, Janssen Cilag, Medtronic, and GE Healthcare. Andrew McEvoy has received support from UCB, Baxter, and Cyberonics. The remaining authors have no conflicts of interest.

This publication presents independent research supported by the Health Innovation Challenge Fund (HICF-T4-275, Programme Grant 97914), a parallel funding partnership between the Department of Health and Wellcome Trust. The views expressed in this publication are those of the author(s) and not necessarily those of the Department of Health or Wellcome Trust.

## References

[B0] Cardinale F (2013). Stereoelectroencephalography: surgical methodology, safety, and stereotactic application accuracy in 500 procedures. Neurosurgery.

[B1] Murphy M, O'Brien TJ, Morris K, Cook MJ (2001). Multimodality image-guided epilepsy surgery. J Clin Neurosci.

[B2] Murphy MA, O'Brien TJ, Morris K, Cook MJ (2004). Multimodality image-guided surgery for the treatment of medically refractory epilepsy. J Neurosurg.

[B3] Harput MV, Gonzalez-Lopez P, Ture U (2014). Three-dimensional reconstruction of the topographical cerebral surface anatomy for presurgical planning with free OsiriX Software. Neurosurgery.

[B4] Duncan JS (2011). Selecting patients for epilepsy surgery: synthesis of data. Epilepsy Behav.

[B5] Duncan JS (2010). Imaging in the surgical treatment of epilepsy. Nat Rev Neurol.

[B6] Cossu M (2005). Stereoelectroencephalography in the presurgical evaluation of focal epilepsy: a retrospective analysis of 215 procedures. Neurosurgery.

[B7] Cossu M (2006). Stereo-EEG in children. Childs Nerv Syst.

[B8] Gonzalez-Martinez J (2014). Stereotactic placement of depth electrodes in medically intractable epilepsy. J Neurosurg.

[B9] Gonzalez-Martinez JA (2012). Stereoelectroencephalography in the ''difficult to localize'' refractory focal epilepsy: Early experience from a North American Epilepsy Center. Epilepsia.

[B10] Rodionov R (2013). Feasibility of multimodal 3D neuroimaging to guide implantation of intracranial EEG electrodes. Epilepsy Res.

[B11] Nowell M (2015). Utility of 3D multimodality imaging in the implantation of intracranial electrodes in epilepsy. Epilepsia.

[B12] Zombori G (2014). Information Processing in Computer-Assisted Interventions.

[B13] Clarkson MJ (2014). The NifTK software platform for image-guided interventions: platform overview and NiftyLink messaging. Int J Comput Assist Radiol Surg.

[B14] Zuluaga MA (2014). Medical Image Computing and Computer-Assisted Intervention-MICCAI. 2014.

[B15] Nowell M (2015). Comparison of computer-assisted planning and manual planning for depth electrode implantations in epilepsy. J Neurosurg.

